# Comparison of Severe Viral Pneumonia Caused by SARS-CoV-2 and Other Respiratory Viruses Among Malaysian Children During the COVID-19 Pandemic

**DOI:** 10.3389/fped.2022.865099

**Published:** 2022-04-25

**Authors:** David Chun-Ern Ng, Kah Kee Tan, Grace Sieng Sing TING, Chin Ling, Nur Fadzreena Binti Fadzilah, Shir Fong TAN, Thayasheri Subramaniam, Nur Emylia Binti Zailanalhuddin, Hui Yi LIM, Suhaila Binti Baharuddin, Yee Lean LEE, Airena Mohamad Nor, Erwin Jiayuan Khoo

**Affiliations:** ^1^Department of Pediatrics, Hospital Tuanku Ja'afar Seremban, Seremban, Malaysia; ^2^Department of Pediatrics, Perdana University-Royal College of Surgeons in Ireland School of Medicine, Seremban, Malaysia; ^3^Microbiology Unit, Department of Pathology, Hospital Tuanku Ja'afar Seremban, Seremban, Malaysia; ^4^Department of Pediatrics, International Medical University, Seremban, Malaysia

**Keywords:** children, COVID-19, SARS-CoV-2, severe pneumonia, viral pneumonia

## Abstract

**Objectives:**

We described the etiology of severe pneumonia in children during the height of the COVID-19 pandemic in Malaysia and compared the clinical features of severe SARS-CoV-2 to other respiratory viruses.

**Methods:**

This retrospective study included all children aged 12 years and below hospitalized with severe pneumonia in Negeri Sembilan, Malaysia, between 1 April 2021 and 31 October 2021. We extracted demographic and clinical data and used logistic regression to examine risk factors associated with severe SARS-CoV-2 or other viral pneumonia.

**Results:**

A total of 111 children were included. The median age was 15 months. Human rhinovirus/enterovirus, SARS-CoV-2 and respiratory syncytial virus were the most common etiology of severe pneumonia. Codetection of >1 viral pathogen was present in 14 (12.6%) patients. Children with severe COVID-19 presented early in the course of illness and had lower rates of pediatric intensive care admission. The presence of sick contact with an adult was a predictor for SARS-CoV-2, whereas adventitious breath sounds were predictive of other respiratory viruses.

**Conclusions:**

The etiology of severe pneumonia in children evolved with the epidemic curve of COVID-19 and school closures. Children with severe pneumonia due to SARS-CoV-2 experienced a milder clinical course when compared to other respiratory viruses.

## Introduction

The coronavirus disease 2019 (COVID-19) pandemic, caused by severe acute respiratory syndrome coronavirus 2 (SARS-CoV-2), is an unprecedented global crisis. Many countries implemented strict control strategies to contain the spread of the pandemic before widespread vaccination coverage was achieved, including lockdowns and school closures and the adoption of non-pharmaceutical interventions (wearing face masks, social distancing measures and travel restrictions). These interventions, which were originally aimed at reducing the impact of the COVID-19 pandemic also affected the transmission dynamics of other viral respiratory infections (1).

Acute respiratory infections are the major cause of morbidity and mortality in young children, especially in developing countries (2). It is of interest to know the etiology of severe pneumonia in children during the COVID-19 pandemic considering the alterations in transmission patterns of respiratory viruses during this period, and its impact on public health control and clinical case management. Therefore, we studied the clinical and epidemiologic aspects of severe viral respiratory infections during the outbreak of COVID-19. We present the findings on circulating viruses causing severe pneumonia in children and the clinical course of COVID-19 in comparison with other respiratory viruses at the height of the COVID-19 pandemic in Malaysia.

## Methods

### Setting

This was a hospital-based, retrospective observational study of children aged ≤ 12 years hospitalized for severe pneumonia between 1 April 2021 and 31 October 2021. The study was performed at Tuanku Ja'afar Seremban Hospital. The hospital is the only tertiary referral center in the state of Negeri Sembilan, Malaysia, serving approximately 1,100,000 people, including 215,000 children aged ≤ 12 years. Children vaccinated under the national immunization program would have received diphtheria, tetanus and pertussis with *Haemophilus influenza b* (Hib) and inactivated poliovirus (IPV) vaccine (DTaP/Hib/IPV) at 2, 3, and 5 months of age. Malaysia has reported high immunization coverage over more than 98% among children (3). Pneumococcal 10-valent conjugate vaccine was introduced into the national immunization program in December 2020, whereas influenza vaccination was not mandatory in the population. This study was performed before COVID-19 vaccinations were available to children below 12 years old in the country.

### Data Collection and Study Definitions

We included all children aged 12 years and below who were admitted to the pediatric respiratory ward or pediatric intensive care unit (ICU) between 1 April 2021 and 31 October 2021 with a diagnosis of severe viral pneumonia or severe SARS-CoV-2 pneumonia. Patients who were tested negative for a viral etiology and patients who had an incomplete virological workup were excluded.

Data were extracted from the patient's electronic medical records into a manual case record form for patients who met the eligibility criteria. Information retrieved included demographic characteristics, comorbidities, immunization status, sick contact, presenting symptoms and their duration, lung auscultation findings, laboratory parameters, treatment received and outcomes.

Severe pneumonia was modified from the World Health Organization's definition (4), as the presence of cough or difficulty breathing with signs of severe respiratory distress, requiring high dependency care in the pediatric respiratory ward or admission to the pediatric ICU. Severe SARS-CoV-2 pneumonia was defined as a patient with confirmed SARS-CoV-2 infection with clinical features of pneumonia requiring supplemental oxygen therapy, according to the local guidelines (5). Patients were admitted to the pediatric ICU if they required inotropes, non-invasive ventilation, mechanical ventilation support, or continuous vital sign monitoring based on the clinician's discretion. All symptoms and signs were collected at the time of presentation. Fever was defined as temperature ≥37.5 C. Duration of illness was calculated from the onset of the first reported symptom. An adult sick contact was defined as a sick contact with an index case above 12 years old, whereas child sick contact was defined as sick contact with an index case below 12 years old. Sick contact was defined as a symptomatic household or social contact (spending face-to-face contact within 1 meter for >15 min) in the preceding 2 weeks prior to the onset of the patient's symptoms. When multiple family members were symptomatic, the first family member who became symptomatic was regarded as the index case. Adventitious breath sounds were defined as the presence of either crackles, wheeze or rhonchi on lung auscultation.

### Laboratory Methods

A nasopharyngeal aspirate or endotracheal aspirate (if ventilated) was obtained from each patient within the first 24 h of admission. Pathogen detection techniques were differentiated based on whether the patients were admitted to the pediatric ICU or the pediatric respiratory ward. Our healthcare system restricted multiplex real-time polymerase chain reaction (RT-PCR) assay testing to the patients in the pediatric ICU to conserve test supplies. The patients admitted to the pediatric ICU were tested with the QIAstat-Dx^®^ Respiratory SARS-CoV-2 Panel (QIAGEN, Germany). The QIAstat-Dx^®^ Respiratory SARS-CoV-2 panel is a multiplex RT-PCR panel that simultaneously detects 22 viral and bacterial respiratory pathogens, including SARS-CoV-2 (E and Orf1 genes), human adenovirus (hAdv), human bocavirus (hBoV), human coronaviruses (229E, HKU 1, NL63, OC43), human metapneumovirus (hMPV), human rhinovirus/enterovirus (hRV/EV), influenza A (H1N1/2009, H1, H3), influenza B virus, parainfluenza viruses (1, 2, 3 and 4), respiratory syncytial virus A/B (RSV-A/B), *Bordetella pertussis, Legionella pneumophilia* and *Mycoplasma pneumoniae*. Patients admitted to the pediatric respiratory ward were tested for SARS-CoV-2 using real-time PCR (Allplex SARS-CoV-2 Assay, Seegene Inc, Republic of South Korea) and other viruses such as RSV, influenza A, influenza B and hAdv using chromatographic immunoassay antigen testing (CerTest Biotech S.L, Zaragoza, Spain).

### Statistical Analysis

Categorical variables were presented as frequency and percentage (%), and continuous variables were presented as median and interquartile range (IQR). Non-parametric two-tailed Mann–Whitney U-test was used for continuous variables and the Chi-squared test or Fisher's exact test was used for categorical variables, as appropriate. Variables with a *p*-value < 0.05 in the univariate analysis were entered into the multivariate logistic regression analysis to identify independent risk factors associated with severe SARS-CoV-2 or other viral pneumonia. All probabilities were two-tailed, and p < 0.05 was considered to indicate statistical significance. Data analysis was performed using SPSS Version 26.0 (IBM Corp., Armonk, NY, USA).

### Ethical Considerations

The study was registered with the National Medical Research Register (NMRR-21-1158-60295) and approved by the Medical Research and Ethics Committee, Ministry of Health Malaysia (KKM/NIHSEC/P21-1195).

## Results

### Baseline Characteristics

**Figure 1:**
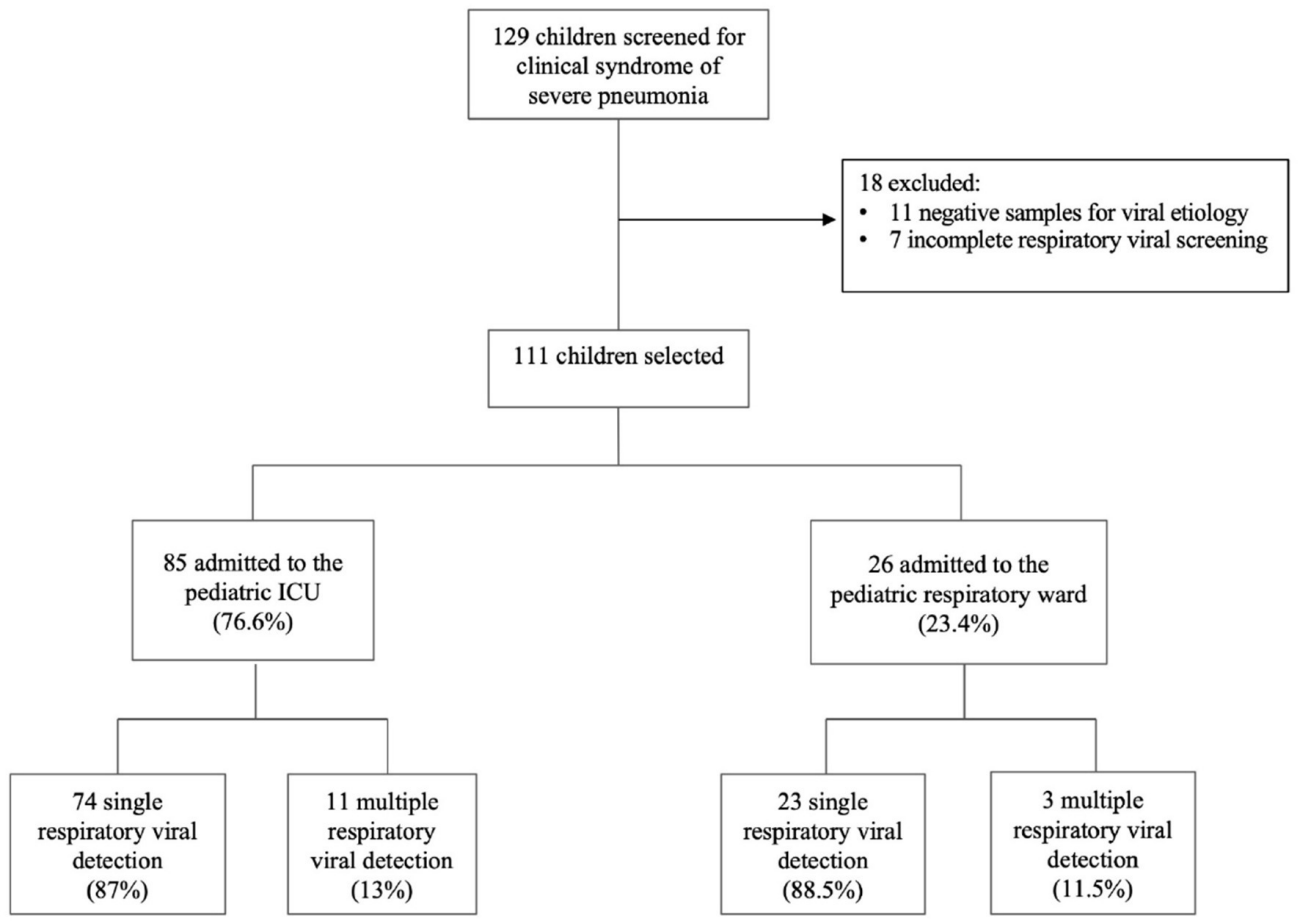
Study flowchart.

A total of 129 patients were diagnosed with severe pneumonia in our institution during the study period. 18 patients were excluded because some had incomplete data while others had negative microbiological investigation results (Figure 1). Eventually, 111 patients were included in the analysis, including 66 males (59.5%) and 45 females (40.5%). The baseline characteristics of the study population are summarized in Table 1. The median age was 15 months (IQR 6-30), with more than two-thirds of patients aged below 24 months. At least one comorbidity was identified in 27 patients (24.3%), with respiratory conditions the most common comorbidity. The number of patients who received at least one dose of Hib vaccination and pneumococcal vaccination was 96 (86.5%) and 42 (37.8%), respectively. Only three patients (2.7%) received influenza vaccination in the preceding 12 months, whereas no patients received Palivizumab in the preceding month before hospitalization. Overall, the median duration of hospitalization was 5 days (IQR 4-8), and all patients were discharged alive.

**Table 1:** Baseline characteristics of the study population.

**Baseline Characteristics of patients**	**Total (*n* = 111)**
Age, months • <12 months • 12–23 months • 24–59 months • 60 months and above	15 (6–30) 49 (44.1%) 28 (25.2%) 25 (22.5%) 9 (8.1%)
Sex	
• Male • Female	66 (59.5%) 45 (40.5%)
Ethnicity	
• Malay • Chinese • Indians • Others	101 (91.0%) 4 (3.6%) 3 (2.7%) 3 (2.7%)
Comorbidities^a^	
• None • Respiratory • Neurological • Cardiovascular • Genetic • Ex-premature infant (if age <2 years old) • Others	84 (75.7%) 15 (13.5%) 7 (6.3%) 5 (4.5%) 5 (4.5%) 3 (2.7%) 3 (2.7%)
Hib vaccination^b^	96 (86.5%)
Pneumococcal vaccination^b^	42 (37.8%)
Influenza vaccination^c^	3 (2.7%)
Palivizumab^d^	0 (0%)
Length of stay, days	5 (4–8)
Outcome	
• Death • Discharged alive	0 (0%) 111 (100%)
Viral etiology (single viral pathogen)	
• Human Rhinovirus/Enterovirus (HRV/EV) • SARS-CoV-2 • Respiratory syncytial virus (RSV) • Human bocavirus (HBoV)	97 (87.4%) 40 (36.0%) 27 (24.3%) 26 (23.4%) 4 (3.6%)
Viral etiology (>1 viral pathogen) • HRV/EV + RSV • HRV/EV + Adenovirus • HRV/EV + SARS-CoV-2 • HRV/EV + Parainfluenza 3 • HRV/EV + HBoV • SARS-CoV-2 + RSV • SARS-CoV-2 + Influenza B • HBoV + Parainfluenza 3	14 (12.6%) 4 (3.6%) 3 (2.7%) 2 (1.8%) 1 (0.9%) 1 (0.9%) 1 (0.9%) 1 (0.9%) 1 (0.9%)

^a^*a patient may have more than 1 comorbidity*.

*^b^defined as received at least 1 dose of vaccine*.

*^c^defined as received influenza vaccine in the preceding 12 months*.

*^d^defined as received at least 1 dose of palivizumab in the preceding month*.

**Figure 2:**
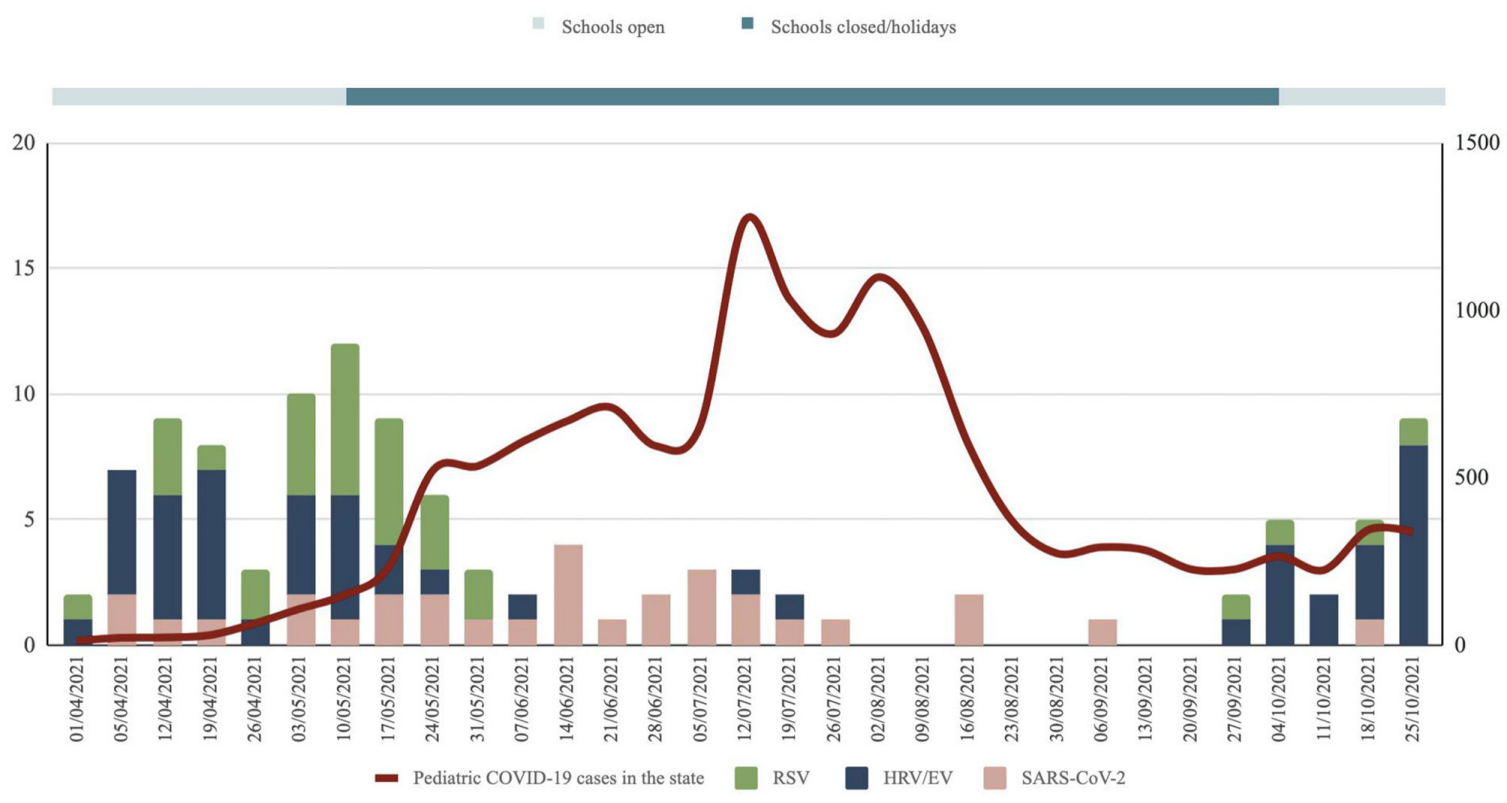
Trends of the three major viruses causing severe pneumonia during the study period in relation to the incidence of pediatric SARS-Cov-2 in the state. Data for pediatric SARS-Cov-2 cases in the state obtained from the state health department.

97 patients (87.4%) had a single viral pathogen detected. Detection of >1 viral pathogen was present in 14 patients (12.6%). The most common single pathogen detected was HRV/EV (*n* = 40, 36.0%), followed by SARS-CoV-2 (*n* = 27, 24.3%) and RSV (*n* = 26, 23.4%). Figure 2 shows the trends of the three major viruses causing severe pneumonia during the study period in relation to the incidence of pediatric SARS-CoV-2 in the state. The etiology of severe pneumonia evolved with the activity of COVID-19 and school closures. SARS-CoV-2 accounted for most of the severe pneumonia cases at the height of the COVID-19 pandemic. However, as COVID-19 dissipates, HRV/EV and RSV resurgence were seen.

The most common dual pathogen combinations were HRV/EV and RSV in 4 patients (3.6%) and HRV/EV and Adenovirus in 3 patients (2.7%). Out of the 14 patients with codetection of multiple viruses, HRV/EV was most commonly present with RSV (4 samples), Adenovirus (3 samples), SARS-CoV-2 (2 samples), and Parainfluenza 3 and HBoV (1 sample each).

### Comparison of Patient Characteristics With Severe Pneumonia Due to SARS-CoV-2 and Other Respiratory Viruses

The median age of patients with severe pneumonia due to SARS-CoV-2 was 17.5 months (IQR 2.0 – 46.9 months) vs. 15.2 months (IQR 6.5 – 26.2 months; *p* = 0.863) for those due to other respiratory viruses. There was no significant difference between gender and underlying comorbidities of both groups. The proportion of patients exposed to a symptomatic adult index was significantly higher in the SARS-CoV-2 group (77.8 vs. 6.3%, *p* < 0.001). In contrast, the proportion of patients who had sick contact with a child was higher in the other respiratory virus group (40 vs. 11.1%, *p* = 0.006) (Table 2).

**Table 2:** Demographics, clinical characteristics and outcomes of patients presenting with severe SARS-CoV-2 compared to other respiratory viruses.

	**Total (*n* = 111)**	**SARS-CoV-2 (*n* = 27)**	Other respiratory viruses*(*n* = 80)	***p*-value**
Age	15.0 (6-30)	17.5 (2.0–46.9)	15.2 (6.5–26.2)	0.863
Male gender	66 (59.5%)	14 (51.9%)	50 (62.5%)	0.329
Comorbidities	27 (24.3%)	8 (29.6%)	19 (23.8%)	0.543
Adult sick contact	29 (26.1%)	21 (77.8%)	5 (6.3%)	<0.001
Child sick contact	35 (31.5%)	3 (11.1%)	32 (40%)	0.006
Duration of illness before hospitalization	2 (2–4)	3 (2–4)	2 (2, 3)	0.479
Fever	81 (73.0%)	21 (77.8%)	58 (72.5%)	0.590
Cough	93 (83.8%)	15 (55.6%)	74 (92.5%)	<0.001
Rhinorrhea	71 (64.0%)	10 (37.0%)	57 (71.3%)	0.001
Vomiting	10 (9.0%)	3 (11.1%)	7 (8.8%)	0.710
Diarrhea	7 (6.3%)	2 (7.4%)	4 (5.0%)	0.641
Seizures	2 (1.8%)	1 (3.7%)	1 (1.3%)	0.443
Rash	3 (2.7%)	0 (0%)	3 (3.8%)	0.570
Anosmia/ ageusia	0 (0%)	0 (0%)	0 (0%)	-
Temperature on arrival, °C	37.5 (36.8–38)	37.3 (36.8- 37.9)	37.6 (36.8- 38.0)	0.610
Shock	4 (3.6%)	0 (0%)	4 (5.0%)	0.570
Adventitious breath sounds	83 (74.8%)	6 (22.2%)	73 (91.3%)	<0.001
Total white cell count, x10^9^/L	11.9 (9.4-14.9)	11.1 (7.3–14.0)	12.4 (9.7–15.5)	0.105
Absolute lymphocyte count, x10^9^/L	3.3 (2.0–5.2)	4.1 (1.9–6.2)	3.0 (2.0–4.8)	0.145
Platelet count, x10^9^/L	335 (278–429)	267 (224–411)	342 (296–430)	0.028
CRP, mg/L	5.2 (0.8–17.0)	1.6 (0.4–14.8)	8.0 (1.9–21.8)	0.152
PICU admission	85 (76.6%)	15 (55.6%)	69 (86.3%)	0.001
HFNC/NIV	69 (62.2%)	7 (25.9%)	61 (76.3%)	<0.001
Mechanical ventilation	19 (17.1%)	0 (100%)	19 (23.8%)	0.003
Duration of oxygen therapy, days	4 (3–7)	2 (1–6)	5 (3–7)	<0.001
Empirical antibiotics	82 (73.9%)	15 (55.6%)	67 (83.8%)	0.003
Steroids	18 (16.2%)	7 (25.9%)	10 (12.5%)	0.128
IV immunoglobulin	4 (3.6%)	0 (0%)	4 (5.0%)	0.570
Inotropes	7 (6.3%)	0 (100%)	7 (8.8%)	0.188
Blood transfusion	11 (9.9%)	1 (3.7%)	10 (12.5%)	0.284
Length of stay, days	5 (4–8)	5 (4–10)	5 (3–8)	0.285

*4 patients who had severe SARS-CoV-2 with co-detection of other respiratory viruses were excluded in this analysis.

*CRP, C-reactive protein; PICU, pediatric intensive care unit; HFNC, high flow nasal cannula; NIV, non-invasive ventilation*.

### Clinical Symptoms and Laboratory Investigations

On admission, cough (83.8.%, 93/111), fever (73%, 81/111), and rhinorrhea (64%, 71/111) were the three most common presenting symptoms for patients presenting with severe pneumonia. 55.6% of patients with severe SARS-CoV-2 had cough, significantly less than patients with other respiratory viruses (92.5%, *p* < 0.001). The proportion of patients with rhinorrhea (37.0%) and adventitious breath sounds (22.2%) was significantly lower in patients with severe SARS-CoV-2 than those with other respiratory viruses (71.3%, *p* = 0.001; 91.3%, *p* < 0.001, respectively). No significant differences were found in the proportion of patients who presented with fever, rash, vomiting or diarrhea (*p* > 0.05). The median duration of illness before hospitalization, the temperature on arrival to hospital, total white count, absolute lymphocyte count, platelet count and C-reactive protein levels did not differ significantly between both groups.

### Treatment and Outcomes

The proportions of patients with severe COVID-19 requiring pediatric ICU admission were lower than those with other respiratory viruses (55.6 vs. 86.3%, *p* = 0.001). Similarly, the proportion of patients who received non-invasive ventilation was lower in the SARS-CoV-2 group (25.9 vs. 76.3%, *p* < 0.001). None of the patients with severe COVID-19 required invasive mechanical ventilation, whereas 23.8% of patients with other respiratory viruses received mechanical ventilation (*p* = 0.003). The median duration of oxygen therapy was significantly lower in the SARS-CoV-2 group (2 days vs. 5 days, *p* < 0.001). 55.6% of patients with severe COVID-19 received antibiotics, significantly lower than the proportion of patients with other respiratory viruses (83.8%, *p* = 0.003). There was no difference in the duration of hospitalization between both groups, and no mortality was reported in both groups.

### Multivariate Analysis of Independent Risk Factors to Differentiate Severe COVID-19 From Other Viral Pneumonia

Patients with severe COVID-19 were more likely to have sick contact with an adult than those with severe pneumonia due to other respiratory viruses (aOR 26.86, 95% CI 5.79–124.76; *p* < 0.001). On the other hand, patients with severe COVID-19 had a lower disposition to exhibit adventitious breath sounds than those with other respiratory viruses (aOR 0.05, 95% CI 0.01–0.26, *p* < 0.001) (Table 3).

**Table 3:** Multivariate analysis of independent risk factors for differentiating severe COVID-19 from other viral Pneumonia.

**Variable**	**Univariate analysis**			**Multivariate analysis**		
	**OR**	**95% CI**	***p*-value**	**OR**	**95% CI**	***p*-value**
Age	1.058	1.009–1.110	0.020	1.029	0.999–1.061	0.057
Male gender	0.270	0.024–3.083	0.292	-	-	-
Adult sick contact	38.515	3.177–466.948	0.004	26.864	5.785–124.756	<0.001
Presence of comorbidities	0.712	0.052–9.814	0.799	-	-	-
Fever	0.994	0.077–12.769	0.994	-	-	-
Cough	0.142	0.007–2.896	0.205	-	-	-
Rhinorrhea	0.961	0.056–16.364	0.978	-	-	-
GI symptoms*	0.204	0.006–7.509	0.388	-	-	-
Adventitious breath sounds	0.033	0.002–0.514	0.015	0.049	0.009–0.259	<0.001
TWC	1.050	0.801–1.377	0.722	-	-	-
ALC	1.290	0.810–2.055	0.283	-	-	-
Platelet count	1.002	0.994–1.010	0.559	-	-	-

*GI symptoms, gastrointestinal symptoms (vomiting or diarrhea).

*TWC, total white cell; ALC, absolute lymphocyte count*.

## Discussion

Our study revealed three major viruses circulating during the COVID-19 pandemic in 2021 which caused severe pneumonia in children, namely HRV/EV, SARS-CoV-2 and RSV. 106 out of the 111 respiratory samples (95.5%) sent detected either one of these three viruses, some in combination with other respiratory viruses. HRV/EV was the most common etiology for severe pneumonia in our population during the study period, accounting for 36.0% of the samples with a single viral pathogen. The etiological role of HRV/EV has been questioned due to high detection rates even in asymptomatic subjects (6, 7). Nevertheless, we deduced HRV/EV as the causative agent for these patients, given the absence of other detectable viral or bacterial pathogens. These results were consistent with findings elsewhere documenting high HRV/EV transmission during the COVID-19 pandemic (8–11).

There were several plausible explanations for the dominance of rhinovirus amongst other respiratory viruses during the COVID-19 pandemic. Many rhinovirus infections are asymptomatic and therefore easily spread as asymptomatic carriers unknowingly transmit the virus (12). In our study, more than half of the patients with severe HRV/EV did not report a sick contact (Supplementary Table), suggesting that there could have been transmission by asymptomatic carriers at home. Additionally, face masks appeared less efficient at filtering rhinoviruses out of exhaled breaths than they are at reducing influenza droplets and seasonal coronavirus aerosols (13). This could contribute to rhinovirus transmission despite universal masking, particularly in adults who develop asymptomatic infection and subsequently transmit it to their children. Furthermore, the non-enveloped rhinoviruses tend to be more environmentally stable and relatively resistant to ethanol-containing disinfectants (14). This could be one of the reasons for the resurgence of rhinovirus cases as the schools reopened due to the greater propensity for contact transmission in addition to droplet transmission.

The etiology of severe pneumonia during the COVID-19 pandemic evolved over the study period (Figure 2). The closure of schools reduced the circulation of common childhood respiratory viruses such as rhinovirus and RSV. During that period, cases of severe pneumonia were caused by SARS-CoV-2. With children confined to their homes, they were most likely infected by adults in the household who had interactions with the community. We observed a shift in the etiology of severe pneumonia from SARS-CoV-2 to HRV/EV as COVID-19 activity came down and schools reopened. The rebound of HRV/EV with the reopening of schools and daycares was well-documented during the COVID-19 pandemic (15, 16). This is understandable because children are the main reservoirs and transmission drivers of rhinovirus (17). Most children experienced an “immunity debt” or a lack of immune stimulation due to reduced exposures with prolonged school closures. This led to a surge of cases as school attendance resumed, compounded by the children's difficulty maintaining social distancing in school.

Notably, only one patient had Influenza (codetection of Influenza B with SARS-CoV-2) despite only 2.7% of the study population who received Influenza vaccination in the preceding year. Our results support previous findings that Influenza activity was low during the COVID-19 pandemic (18, 19). Although empirical antibiotics were started for 73.9% of cases with severe pneumonia, none of the patients' blood cultures were positive. There were no invasive pneumococcal coinfections despite the low pneumococcal vaccination rates among the study population. The low incidence of *Streptococcus pneumoniae* during the COVID-19 pandemic likely contributed to this (20).

We observed that severe COVID-19 in children exhibits different characteristics compared to the classical adult-type disease described in literature. Severe COVID-19 in adults is characterized by a hyperinflammatory response that typically occurs in the second week of illness with abnormal biomarkers such as lymphopenia and raised C-reactive protein (CRP) levels (21, 22). In contrast, our patients with severe COVID-19 presented early in the course of illness (median 3 days after symptom onset), had no lymphopenia (median absolute lymphocyte count, ALC 4.1 x 10^9^/L) and normal CRP levels (median CRP 1.6 mg/L). These findings were consistent with studies that had observed children were hospitalized at a median of 2 days from onset of symptoms (23) and were less likely to exhibit a profound inflammatory response even in severe disease (24). The relatively mild clinical course of COVID-19 in children is somewhat unusual, considering that other viral respiratory infections such as RSV or Influenza are generally more severe in children. The differences in disease progression between children and adults are not fully understood but are likely a combination of factors that place adults at higher risk for severe disease and those that protect children (25). Patients hospitalized for multisystem inflammatory syndrome (MIS-C) were not part of the inclusion criteria, although some required admission to the pediatric ICU as we decided to focus on acute respiratory infections in children. Furthermore, many of our patients with MIS-C did not have significant respiratory system involvement.

Although fever, cough and rhinorrhea were the most common symptoms of children with severe COVID-19, we observed that the proportion of patients with cough and rhinorrhea was significantly lower than that of other respiratory viruses. The presence of cough was only reported in around one-third of children admitted to the ICU in a previous study (26). In our study, COVID-19 results in less severe disease when compared to other respiratory viruses, evidenced by the lower proportion of patients who required ICU admission, high flow nasal cannula/non-invasive ventilation, mechanical ventilation and empirical antibiotics. Additionally, the duration of oxygen therapy was significantly shorter among patients with SARS-CoV-2 compared to other respiratory viruses. The presence of an adult sick contact was a predictor for SARS-CoV-2, whereas adventitious breath sounds in a child with severe pneumonia were predictive of other respiratory viruses as the etiology.

This study has several important clinical implications. Firstly, children with severe COVID-19 presented early in the course of illness without abnormal biomarkers such as lymphopenia and raised CRP. This suggests that the pathophysiology of severe COVID-19 in children is different from adults, where a transition to a hyperinflammatory state could occur during later stages. Although the usage of corticosteroids has been shown beneficial in hospitalized adults requiring respiratory support (27), our study showed that routine corticosteroids for pediatric COVID-19 are not required even in those who require respiratory support. In our study, only 25.9% of children with severe SARS-CoV-2 received corticosteroids during hospitalization. Secondly, 4/31 (12.9%) of patients with severe SARS-CoV-2 had codetection of other respiratory pathogens, namely RSV (two patients), Influenza B (1 patient) and Parainfluenza 3 (one patient). Since SARS-CoV-2 is generally a milder disease when compared to other respiratory pathogens, attempts to search for coinfections with other respiratory pathogens should be made when a child presents with severe pneumonia due to SARS-CoV-2. The presence of coinfections of SARS-CoV-2 with other respiratory pathogens was as high as 33–51% in previous studies (28, 29). Thirdly, we observed that in a child who presents with severe pneumonia, the presence of adventitious breath sounds with a child sick contact suggests a non-SARS-CoV-2 etiology. This is understandable as HRV/EV was the predominant circulating virus during the study period, and children are important reservoirs of these viruses. On the other hand, adults were the primary source of household transmission of SARS-CoV-2 as shown in a local study (30), and sick contact with an adult was a predictor for SARS-CoV-2.

Our findings are subject to several limitations. The respiratory virus detection method was not standardized for all patients. Multiplex PCR respiratory viral panel (RVP) testing was only available for patients admitted to the pediatric ICU due to budget limitations. The usage of chromatographic immunoassays with lower sensitivity and a limited panel of pathogens would affect our codetection rates. Our viral codetection rates could have been potentially higher if multiplex PCR RVP assays had been used for all cases. Nevertheless, our codetection rate of 12.6% (14/111) was higher than other studies using multiplex PCR panel assays (8), suggesting that most of our patients likely had a single pathogen infection even if the respiratory viral panel was used universally for all. Patients with a negative or incomplete virological workup who were omitted from the final analysis could have had severe pneumonia of another etiology. While this omission allowed us to compare the clinical features of patients with a confirmed viral etiology, it could have introduced ascertainment bias when comparing the clinical features of patients with severe SARS-CoV-2 and those with severe pneumonia of other etiology. Further details of the patients who were omitted were available in the supplementary table. Additionally, being a retrospective study, we did not have complete data for other laboratory parameters such as procalcitonin, serum transaminases and arterial blood gas analysis as these investigations were not routinely carried out for all cases. We did not collect data on chest radiography results as radiological reporting for the chest radiographs was not routinely performed at the height of the COVID-19 pandemic. The documented symptoms and laboratory results were the baseline upon hospital admission, and this study did not capture longitudinal data regarding the development of new symptoms or changes in laboratory parameters. Lastly, genomic sequencing for SARS-CoV-2 variants and molecular studies to distinguish the various species of HRV/EV were not performed. Nevertheless, the study period coincides with the peak of the COVID-19 epidemic in Malaysia, as the Delta variant displaced Beta and became the dominant strain in the country (31). Despite those limitations, this study provides a sufficiently broad overview of the etiology and clinical features of severe pneumonia in children during the height of the COVID-19 pandemic in Malaysia.

## Conclusion

In conclusion, our study showed three major viruses causing severe pneumonia in children during the COVID-19 pandemic, namely HRV/EV, SARS-CoV-2 and RSV. COVID-19 pneumonia in children appeared less severe than pneumonia caused by other respiratory viruses circulating during the period. The presence of coinfections with other respiratory viruses should be considered in a child who presents with severe pneumonia due to SARS-CoV-2. Children with severe COVID-19 presented early in the course of illness without any biochemical evidence of hyperinflammation. Lastly, the epidemiological characteristics of respiratory viral pathogens, especially HRV/EV should be evaluated more intensely with the reopening of schools and increased social interaction among children.

## Data Availability Statement

The raw data supporting the conclusions of this article will be made available by the authors, without undue reservation.

## Ethics Statement

The studies involving human participants were reviewed and approved by Medical Research and Ethics Committee, Ministry of Health Malaysia (KKM/NIHSEC/P21-1195). Written informed consent from the participants' legal guardian/next of kin was not required to participate in this study in accordance with the national legislation and the institutional requirements.

## Author Contributions

DN, KT, and SB made substantial contributions to the conception and design of the work. GT, CL, NF, TS, ST, NZ, and HL made substantial contribution to data collection and data analysis. DN, KT, GT, CL, NF, TS, ST, NZ, HL, SB, YL, AM, and EK made substantial contributions to the interpretation of data for the manuscript. DN and EK drafted the initial manuscript. DN, KT, YL, AM, and EK supported literature review. All authors reviewed and revised the manuscript for important intellectual content, giving approval of the version to be published, and gave their agreement to be accountable for all aspects of the work.

## Funding

Open access publication fees are funded by the International Medical University.

## Conflict of Interest

The authors declare that the research was conducted in the absence of any commercial or financial relationships that could be construed as a potential conflict of interest.

## Publisher's Note

All claims expressed in this article are solely those of the authors and do not necessarily represent those of their affiliated organizations, or those of the publisher, the editors and the reviewers. Any product that may be evaluated in this article, or claim that may be made by its manufacturer, is not guaranteed or endorsed by the publisher.
