# Supplementary material for: Comparison of Severe Viral Pneumonia Caused by SARS-CoV-2 and Other Respiratory Viruses Among Malaysian Children During the COVID-19 Pandemic
Source: Front Pediatr. 2022 Apr 25;10:865099. doi: 10.3389/fped.2022.865099 (PMC9082799; doi:10.3389/fped.2022.865099)
Supplement: Supplementary file 1 [file Table_1.pdf]

Supplementary table : Data on the clinical features and outcomes of patients with HRV/EV, RSV, Bocavirus, codetection of >1 virus and excluded patients due to incomplete/ negative virological workup

	Total (n=111)	HRV/EV (n=40)	RSV (n= 26)	HBoV (n=4)	Co-detection >1 virus (n=14)	Excluded patients (n=18)
**Age**	15.0<br>(6-30)	17.5<br>(11.1 – 32.6)	8.4<br>(1.6 - 17.2)	21.7<br>(18.7-27.0)	15.7<br>(4.8 – 30.9)	8.3<br>(1.7-29.8)
**Male gender**	66 (59.5%)	25 (62.5%)	16 (61.5%)	2 (50.0%)	9 (64.3%)	9 (50.0%)
**Comorbidities**	27 (24.3%)	12 (30%)	4 (15.4%)	2 (50.0%)	1 (7.1%)	10 (55.6%)
**Adult sick contact**	29 (26.1%)	2 (5.0%)	2 (7.7%)	1 (25.0%)	3 (21.4%)	2 (11.1%)
**Child sick contact**	35 (31.5%)	16 (40.0%)	12 (46.2%)	0 (0%)	4 (28.6%)	4 (22.2%)
**Duration of illness before hospitalization**	2 (2-4)	2 (2-3)	3 (2-5)	3 (2-4)	2 (2-4)	2 (1-3)
**Fever**	81 (73.0%)	26 (65.0%)	22 (84.6%)	3 (75.0%)	9 (64.3%)	12 (66.7%)
**Cough**	93 (83.8%)	38 (95.0%)	24 (92.3%)	2 (50.0%)	14 (100%)	16 (88.9%)
**Rhinorrhea**	71 (64.0%)	34 (85.0%)	14 (53.8%)	1 (25.0%)	12 (85.7%)	8 (44.4%)
**Vomiting**	10 (9.0%)	4 (10.0%)	1 (3.8%)	1 (25.0%)	1 (7.1%)	5 (27.8%)
**Diarrhea**	7 (6.3%)	4 (10.0%)	0 (0%)	0 (0%)	1 (7.1%)	2 (11.1%)
**Seizures**	2 (1.8%)	0 (0%)	0 (0%)	1 (25.0%)	0 (0%)	0 (0%)
**Rash**	3 (2.7%)	1 (2.5%)	1 (3.8%)	0 (0%)	1 (7.1%)	1 (5.6%)
**Anosmia/ ageusia**	0 (0%)	0 (0%)	0 (0%)	0 (0%)	0 (0%)	0 (0%)
**Temperature on arrival, °C**	37.5<br>(36.8 – 38)	37.6<br>(36.7 - 37.9)	37.7<br>(36.6-38.1)	38.3<br>(37.9-39.1)	37.0<br>(36.9 – 37.4)	37.4<br>(36.6-37.9)
**Shock**	4 (3.6%)	3 (7.5%)	1 (3.8%)	0 (0%)	0 (0%)	0 (0%)
**Adventitious breath sounds**	83 (74.8%)	38 (95.0%)	21 (80.8%)	4 (100%)	14 (100%)	16 (88.9%)
**Total white cell count, x10^9/L**	11.9<br>(9.4-14.9)	13.7<br>(10.5 – 16.5)	9.6<br>(6.8- 13.1)	11.9<br>(10.1-14.0)	13.3<br>(9.5 – 16.1)	12.2<br>(9.1-16.6)
**Absolute lymphocyte count, x10^9/L**	3.3<br>(2.0 – 5.2)	3.0<br>(1.8 – 4.1)	4.3<br>(2.8 – 5.1)	2.1<br>(0.5-5.0)	4.3<br>(2.4 – 6.5)	4.3<br>(3.8-5.8)
**Platelet count, x10^9/L**	335<br>(278- 429)	339<br>(304 – 428)	322<br>(286 – 476)	278<br>(183-453)	379<br>(305 – 452)	406<br>(299- 488)
**CRP, mg/L**	5.2<br>(0.8 – 17.0)	13.0<br>(2.4 – 22.4)	2.5<br>(0.1 – 21.7)	3.6<br>(1.2 – 6.4)	13.9<br>(5.2 – 26.7)	13.6<br>(2.6 – 34.3)
**PICU admission**	85 (76.6%)	38 (95.0%)	17 (65.4%)	4 (100%)	11 (78.6%)	11 (61.1%)
**HFNC/NIV**	69 (62.2%)	30 (75.0%)	21 (80.8%)	2 (50.0%)	9 (64.3%)	16 (88.9%)
**Mechanical ventilation**	19 (17.1%)	10 (25.0%)	5 (19.2%)	2 (50.0%)	2 (14.3%)	2 (11.1%)
**Duration of oxygen therapy, days**	4 (3-7)	4 (3-6)	7 (5 – 9)	5 (3-7)	4 (2-6)	4 (3-8)
**Empirical antibiotics**	82 (73.9%)	34 (85.0%)	20 (76.9%)	4 (100%)	9 (64.3%)	15 (83.3%)
**Steroids**	18 (16.2%)	7 (17.5%)	1 (3.8%)	0 (0%)	3 (21.4%)	3 (16.7%)
**IV immunoglobulin**	4 (3.6%)	2 (5.0%)	1 (3.8%)	0 (0%)	1 (7.1%)	0 (0%)
**Inotropes**	7 (6.3%)	4 (10.0%)	2 (7.7%)	0 (0%)	1 (7.1%)	0 (0%)
**Blood transfusion**	11 (9.9%)	5 (12.5%)	3 (11.5%)	0 (0%)	2 (14.3%)	2 (11.1%)
**Length of stay, days**	5 (4-8)	4 (3-6)	8 (5-10)	6 (4-9)	4 (3-6)	7 (4-13)

HRV/EV = Human rhinovirus/enterovirus, RSV = Respiratory syncytial virus, HBoV = Human bocavirus
CRP = C-reactive protein, PICU = pediatric intensive care unit, HFNC = high flow nasal cannula, NIV = non-invasive ventilation
